# Impact of SARS-CoV-2 Resistance to Antiviral Monoclonal Antibody Therapy on Neutralizing Antibody Response

**DOI:** 10.20411/pai.v9i2.718

**Published:** 2024-08-21

**Authors:** Marc-Kendy Paul, Manish C. Choudhary, Amy L. Heaps, Rinki Deo, Daniela Moisi, Kelley C. Gordon, John W. Mellors, Carlee Moser, Paul Klekotka, Alan Landay, Judith S. Currier, Joseph J. Eron, Kara W. Chew, Davey M. Smith, Scott F. Sieg, Urvi M. Parikh, Jonathan Z. Li, on behalf of the ACTIV-2/A5401 Study Team

**Affiliations:** 1 Brigham and Women’s Hospital, Harvard Medical School, Cambridge, MA; 2 University of Pittsburgh School of Medicine, Pittsburgh, PA; 3 Case Western Reserve University, Cleveland, OH; 4 Harvard T.H. Chan School of Public Health, Boston, MA; 5 Eli Lilly and Company, San Diego, CA; 6 RUSH Medical College, Chicago, IL; 7 David Geffen School of Medicine, University of California, Los Angeles, CA; 8 University of North Carolina at Chapel Hill, Chapel Hill, NC; 9 University of California, San Diego, CA; 10 Case Western Reserve University and University Hospitals Cleveland, Cleveland, OH

**Keywords:** bamlanivimab, COVID-19, monoclonal antibody, SARS-CoV-2, pseudovirus neutralization

## Abstract

**Background:**

Anti-SARS-CoV-2 monoclonal antibodies (mAbs) have played a key role as an anti-viral against SARS-CoV-2, but there is a potential for resistance to develop. The interplay between host antibody responses and the development of monoclonal antibody (mAb) resistance is a critical area of investigation. In this study, we assessed host neutralizing antibody (nAb) responses against both ancestral virus and those with treatment-emergent E484K bamlanivimab resistance mutations.

**Methods:**

Study participants were enrolled in the ACTIV-2/Advancing Clinical Therapeutics Globally (ACTG) A5401 phase 2 randomized, placebo-controlled trial of bamlanivimab 700 mg mAb therapy (NCT04518410). Anterior nasal and nasopharyngeal swabs were collected for SARS-CoV-2 RNA testing and S gene next-generation sequencing to identify the E484K bamlanivimab resistance mutation. Serum nAb titers were assessed by pseudovirus neutralization assays.

**Results:**

Higher baseline (pre-treatment) nAb titers against either ancestral or E484K virus was associated with lower baseline viral load. Participants with emerging resistance had low levels of nAb titers against either ancestral or E484K nAb at the time of study entry. Participants with emergent E484K resistance developed significantly higher levels of E484K-specific nAb titers compared to mAb-treated individuals who did not develop resistance. All participants who developed the E484K mAb resistance mutation were eventually able to clear the virus.

**Conclusion:**

Emerging drug resistance after SARS-CoV-2-specific mAb therapy led to a heightened host neutralizing antibody response to the mAb-resistant variant that was associated with eventual viral clearance. This demonstrates the interplay between the antiviral treatment-directed viral evolution and subsequent host immune response in viral clearance.

## INTRODUCTION

Antiviral monoclonal antibodies (mAbs) have played a key role as an antiviral against SARS-CoV-2 and other viruses [1–7]. Five mAb strategies (bamlanivimab, bamlanivimab/etesevimab, casirivimab/imdevimab, sotrovimab, and bebtelovimab) received emergency authorization from the US Food and Drug Administration for treatment of outpatients and were commonly in use. These mAbs target the Spike protein and block their interaction with the host ACE2 receptor. They were found to be effective in preventing disease progression for non-hospitalized individuals who are at high risk of severe symptoms [3–5, 8]. However, resistance to mAbs can occur quickly in those who are treated, which may lead to rebound of infectious mAb-resistant virus [9]. For example, the E484K escape mutation can develop shortly after bamlanivimab infusion, leading to reduced antibody binding affinity and loss of mAb activity [9–11].

It is unknown how host antibody responses may affect the development of mAb resistance and whether pre-existing and developing antibodies against the resistant strain may impact the emergence and clearance of resistant variants. In this study, we used samples collected from the ACTIV-2 phase 2 placebo-controlled bamlanivimab outpatient treatment trial of individuals with and without emerging bamlanivimab resistance. We assessed neutralizing antibody (nAb) responses against both ancestral virus and virus harboring the E484K bamlanivimab resistance mutation, both at the time of study entry (study day 0/immediately prior to infusion) and 28 days after treatment (study day 28). We evaluated differences in nAb titers between those with and without emerging mAb resistance and assessed the relationship between nAb titers and quantitative SARS-CoV-2 RNA load. The overall goal was to gain insights into the interplay between viral replication, antibody response, and treatment outcomes.

## METHODS

### Study Participants and Sample Collection

The study participants were enrolled in the ACTIV-2/Advancing Clinical Therapeutics Globally (ACTG) A5401 phase 2 randomized, placebo-controlled trial of bamlanivimab 700 mg mAb therapy (NCT04518410) [12]. The protocol was approved by a central institutional review board (IRB), Advarra (Pro00045266), with additional local IRB review and approval as required by participating sites. All participants provided written informed consent. Symptomatic adults ≥18 years of age with a documented positive SARS-CoV-2 antigen or nucleic acid test and without the need for hospitalization were enrolled if the diagnostic sample was collected ≤7 days before study entry and within 10 days of symptom onset. We assessed neutralizing antibody levels at study entry (day 0) and day 28 for mAb-treated participants who had emerging E484K resistance mutations of E484K and comparator groups of mAb-treated participants who did not develop the E484K resistance mutations and placebo-treated participants who were randomly selected amongst those with available samples.

### SARS-CoV-2 Viral Load Testing and S Gene Next-Generation Sequencing

Longitudinal anterior nasal (AN) and nasopharyngeal swab (NP) sampling was performed on study days 0 (study entry, baseline), 3, 7, and 14. These samples were taken for SARS-CoV-2 viral load and for next-generation sequencing of the S gene to evaluate the emergence of resistance. SARS-CoV-2 viral loads from AN swab samples were quantified using the Abbott m2000 system with lower limit of quantification (LLoQ) and limit of detection (LoD) of 2 log_10_ and 1.4 log_10_ copies/mL, respectively. SARS-CoV-2 quantitative Laboratory Developed Test (LDT) was developed utilizing open mode functionality on m2000sp/rt (Abbott) by using EUA Abbott SARS-CoV-2 qualitative reagents described elsewhere [13]. S gene sequencing was performed on nasopharyngeal swab sample for all participants with a SARS-CoV-2 RNA level ≥2 log_10_ SARS-CoV-2 RNA copies/mL at study entry or the earliest subsequent time point. Viral RNA extraction was performed on 1mL of swab fluid by use of the TRIzol-LS™ Reagent (ThermoFisher), as previously described [14]. cDNA synthesis was performed using Superscript IV reverse transcriptase (Invitrogen), and S gene amplification was performed using a nested PCR strategy with in-house designed primer sets targeting codons 1-814 of Spike [9]. NGS library construction was performed using the Nextera XT Library Prep Kit (Illumina). Subsequent sequencing was performed on the Illumina MiSeq platform. Deep sequencing data analysis was carried out using the Stanford Coronavirus Antiviral & Resistance Database (CoV-RDB) platform [15]. Input FASTQ sequence alignment with Wuhan-Hu-1 reference was done using MiniMap2 version 2.22 in CodFreq pipeline (https://github.com/hivdb/codfreq). The output of MiniMap2, an aligned SAM file, was converted to a CodFreq file by an in-house written Python script using a PySam library (version: 0.18.0) and further analyzed with the CoVDB. PCR and sequencing runs were performed once with appropriate positive and negative controls.

### Pseudovirus Neutralization Assays

SARS-CoV-2 pseudovirus (PSV) was generated in 293T cells by co-transfection of pF-C37K-CMV-S (a generous gift from Bill Chen) [16], an enhanced expression plasmid encoding for codon-optimized full-length SARS-CoV-2 S (Wuhan-1 sequence containing D614G substitution) with the N-term HiBit tag removed (or other plasmid containing alternate substitutions), and pNL4-3.luc.R-E-mCherry-luciferase, an envelope-deficient HIV-1 dual reporter construct that was cloned by recombination of the pNL.luc.R-E-plasmid (NIH AIDS Reagent Program) and the fully infectious pNL4-3 mCherry luciferase plasmid (Addgene). After harvest, PSV was centrifuged at 400xg for 10 minutes at room temperature (RT) and supernatant removed and filtered with a 0.45-micron syringe filter to remove producer cells. For neutralization assays, 104 293T-hACE2 cells were plated in 100μL media per well in 96 well white-wall, white-bottom plates (Perkin Elmer) and incubated overnight at 37°C. Sera was diluted 1:1.5, then serially diluted 3-fold and incubated with 50μL of diluted PSV for 1 hour at 37°C. After incubation, media was removed from wells containing 293T-hACE2 cells and replaced with PSV/sera, and spinoculation was performed at 1,000xg for 1 hour at RT. Plates were then incubated for 48 hours at 37°C. After 48 hours, plates were analyzed for luciferase production by adding 100μL of BriteLite Plus reagent (Perkin Elmer), incubating at RT for 2 minutes, and reading on a Victor Nivo microplate luminometer (Perkin Elmer). Results are reported as the highest serum dilution that neutralizes >50% of the PSV, termed the NT50.

### Statistical Analysis

SARS-CoV-2 RNA levels below the LoD were imputed as 0.7 log_10_ copies/mL, while detectable values below the LLoQ were imputed as 1.7 log_10_ copies/mL. Continuous variables are presented as medians with inter-quartile range, while categorical variables are expressed as frequencies or percentages. Non-parametric Wilcoxon rank sum testing was used to assess differences between groups, while the Spearman test was used for analyzing correlations. All statistical analyses were performed in GraphPad Prism (Version 9.1.1).

## RESULTS

We had previously identified 5 participants who had received bamlanivimab 700mg in ACTIV-2 with treatment-emerging E484K resistance mutations [9]. In this study, we assessed the presence of host serum nAb titers against pseudovirus containing Spike with and without E484K resistance mutations in 3 groups of participants: mAb-treated individuals with emerging E484K (N=5), mAb-treated individuals without emerging E484K (N=13), and placebo recipients (N=19). Participants in the study, whether in the placebo group or those with and without the emerging E484K resistance mutation, showed similar demographics, symptom scores, and duration of symptoms at enrollment (Table 1). However, those with the emerging E484K mutation were older and presented with a higher baseline viral load of AN SARS-CoV-2 (Table 1).

**Table 1.** Demographic Characteristics of Participants Categorized in Treatment and Placebo Arms

Characteristic	Treatment (N=18)		Placebo (N=19)
	Participants with emerging E484K mutation (N=5)	Participants without emerging E484K mutation (N=13)	
Median age (Q1, Q3), y	62 (58, 65)	49 (30, 55)	44 (36, 51)
Female sex, %	40	39	47
Race/ethnicity, %			
White	60	85	68
Non-White	40	15	32
Median days from symptom onset to enrollment (Q1, Q3)	5 (4, 8)	4 (3, 5)	6 (4.5, 7)
Symptom score at enrollment (study day 0) (Q1, Q3)	9 (9, 15)	9 (8, 12)	10 (6, 14)
Median AN SARS-CoV-2 viral load at enrollment (Q1, Q3), log_10_ copies/mL	6.76 (5.89, 8.00)	5.77 (3.15, 6.80)	4.79 (3.08, 7.08)

AN = anterior nasal; Q = quartile; y = years.

**Figure 1.**
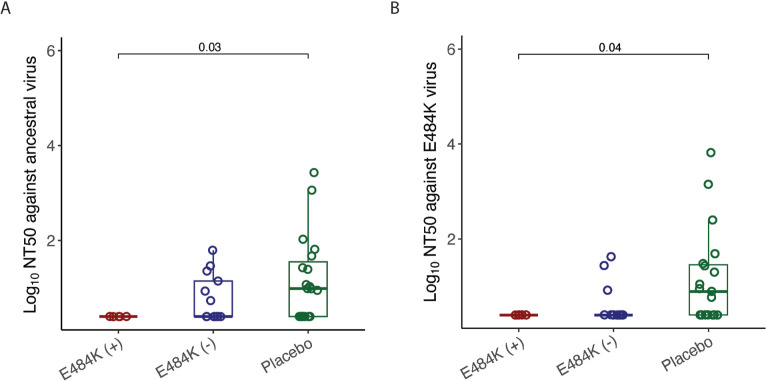
**Comparison of log_10_ NT50 values in different study groups at study entry.** (A) against ancestral virus, (B) against E484K virus. The boxplots represent the 25th and 75th percentiles (bottom and top edge of the box), while the open circles represent individual data point. Median values are represented by horizontal line within the boxplot. Comparison of NT50 values across study groups were compared using Wilcoxon rank sum test. *P*<0.05 were considered statistically significant.

None of the participants with emerging E484K resistance had detectable levels of nAb titers against ancestral virus at the time of study entry, while 46% of mAb-treated participants without E484K and 63% of placebo recipients had detectable nAb titers at baseline against ancestral virus (Figure 1A). Similarly, 13% of mAb-treated participants without E484K and 58% of placebo recipients had detectable nAb titers at baseline against E484K containing virus (Figure 1B). Participants who developed E484K resistance had lower baseline ancestral virus-specific nAb (*P*=0.03) and E484K containing virus (*P*=0.04) levels compared to placebo (Figure 1).

**Figure 2.**
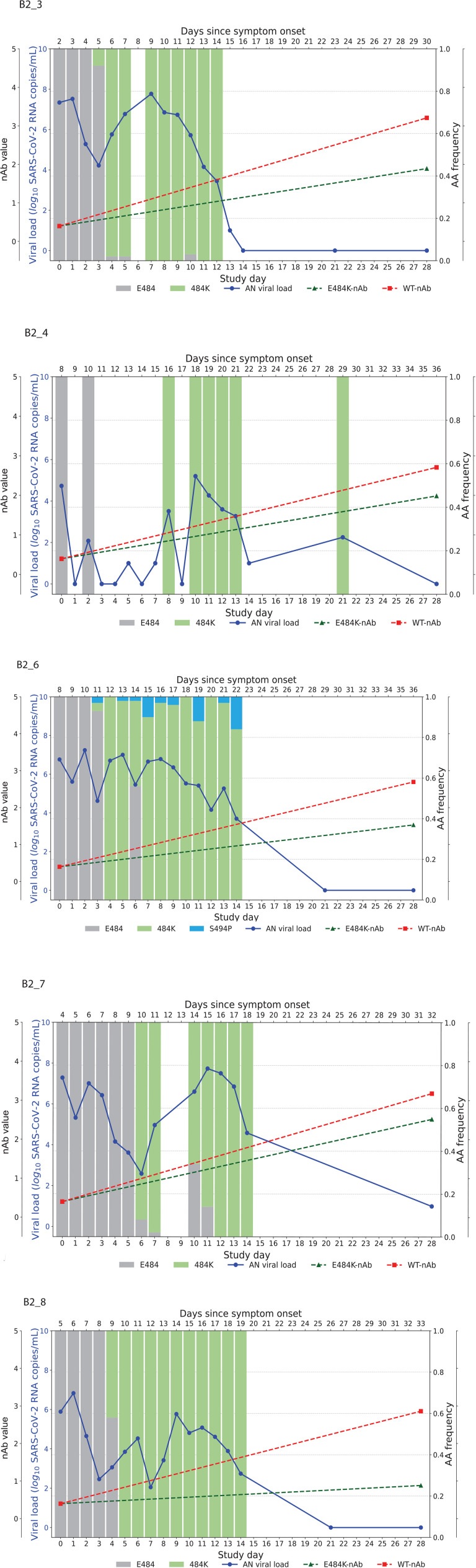
**Figures illustrate the viral loads and frequencies of primary resistance mutations from anterior nasal swab (AN) samples in 5 different participants exhibiting primary bamlanivimab resistance mutations.** The wild-type and resistance mutations are represented by colored bars, indicating different types of changes. The SARS-CoV-2 viral load is depicted with a solid blue line. Neutralizing antibody (nAb) values at baseline and on day 28 for the wild-type and E484K mutations are shown with green and red dotted lines, respectively. The outer and inner left y-axes represent nAb values and viral load in log_10_ copies/mL, while the right y-axis indicates amino acid frequency.

Higher baseline (pre-treatment) nAb titers against either ancestral or E484K virus was associated with lower baseline viral load (ancestral virus: Spearman r = −0.37, *P*=0.022; E484K-containing virus: r = −0.33, *P*=0.042; Supplemental Figure 1). The development of the resistance to bamlanivimab was associated with prolonged viral shedding [9] that was subsequently cleared in all 5 participants (Figure 2). The emergence of SARS-CoV-2 resistance mutations was closely linked to consistent changes in viral load dynamics. This is illustrated in Figure 2, which shows 5 examples (participants B2_3, B2_4, B2_6, B2_7, and B2_8) of viral rebound in participants with the appearance of escape variants. For instance, in participant B2_3, the E484K resistance mutation emerged on study day 3 as a low-frequency variant and quickly became the dominant strain by the following day (Figure 2, top left panel). This mutation was associated with a 3.6 log_10_ copies/mL increase in AN swab viral loads over the next 4 days, reaching a peak of 7.8 log_10_ copies/mL on study day 7 before declining. Similar trends were observed in participants B2_4, B2_6, B2_7, and B2_8. In all the cases, the emergence of bamlanivimab-specific resistance was linked to viral rebound, which was subsequently cleared by day 28 (Figure 2).

**Figure 3.**
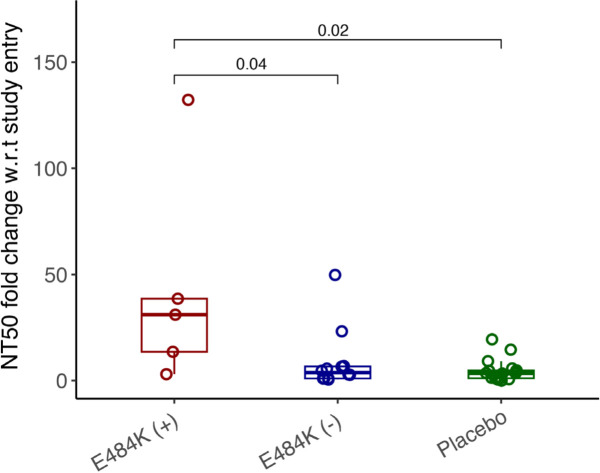
**Comparison of NT50 fold change w.r.t. study entry against E484K virus in different study groups.** Boxplot shows NT50 fold change at day 28 w.r.t. study entry in different study groups against E484K virus. The boxplots represent the 25th and 75th percentiles (bottom and top edge of the box), while the open circles represent individual data point. Median values are represented by horizontal line within the boxplot. Comparison of median values across study groups were compared using Wilcoxon rank sum test. *P*<0.05 were considered statistically significant.

By study day 28, all participants had increasing nAb titers. However, mAb-treated participants had significantly higher nAb titers against ancestral Spike compared to placebo recipients, regardless of resistance emergence, which is likely a reflection of residual bamlanivimab activity (Supplemental Figure 2A). Interestingly, mAb-treated participants in the E484K emerging resistance group not only had evidence of viral rebound, but also developed higher levels of E484K-specific nAb titers (Figure 2, Supplemental Figure 2B) and a significantly greater increase in NT50 fold change at day 28 compared to study enrollment than those without the resistance mutation or those receiving placebo (median fold change for mAb-treated with E484K vs mAb-treated without E484K: 31.1 vs 3.8, *P*=0.04; mAb-treated with E484K vs placebo: 31.1 vs 3.5, *P*=0.02, Figure 3). These results are consistent with a heightened immune response towards the rebounding resistant variants that was associated with the eventual clearance of the mAb resistant variant (Figure 2).

## DISCUSSION

Monoclonal antibodies represented the first antiviral therapy for COVID-19 and were a critical part of early strategies in preventing severe disease and death. In addition, monoclonal antibody treatment remains a key therapeutic option for several other viral infections [17, 18]. We have previously shown that resistance against mAbs can occur in a subset of treated individuals and that this can lead to viral and symptom rebound [9], but factors associated with clearance of mAb-resistant variants remain unclear. In this study, we found that individuals with emerging E484K resistance mutation did not demonstrate significant nAb response against the E484K variant at baseline, but subsequently developed E484K-specific nAb activity in the setting of viral clearance for all individuals harboring resistant variants.

Neutralizing antibody levels against SARS-CoV-2 has been found to be a correlate of protection for vaccines and a crucial aspect of immune-based clearance of infection [19, 20]. Monoclonal antibodies against SARS-CoV-2 represents another success of antibody-mediated viral clearance as bamlanivimab and other mAbs have led to more rapid viral decay and recovery [3]. However, drug resistance against mAbs can occur rapidly in a subset of patients [9, 21]. Our results show that higher levels of nAb at baseline was associated with lower SARS-CoV-2 RNA levels at entry, which is a reflection that in a subset of participants, existing host immune response at the time of study entry was already altering viral kinetics. In addition, the presence of a greater breadth of nAb responses (detectable against both wild-type and E484K resistance variants) was relatively rare at baseline in this high-risk population of adults treated early in their disease course, demonstrating a permissive environment for resistance emergence.

Emergence of E484K resistance mutations was associated with persistent viral shedding, followed by eventual clearance of the resistant variants. This rebound in viral loads at the time of resistance emergence persisted for several days to over 1 week before beginning to decline. In mAb-treated individuals with emerging E484K resistance, the fold-change of E484K variant-specific neutralizing antibodies between day 28 and study enrollment was significantly higher compared to either mAb-treated individuals who did not develop E484K or placebo recipients. These results are consistent with the refocusing of Ab responses against breakthrough variants that then lead to their eventual clearance.

Limitations of this manuscript include a limited sample size of individuals who developed mAb resistance. While mAbs are not currently used as a treatment modality for SARS-CoV-2, pemivibart has been authorized for pre-exposure prophylaxis for COVID-19, and there is currently development of more mAbs for both SARS-CoV-2 and other viruses [17, 18]. These results are important to assess risk factors for development of resistance and provide insights on the role of nAb in natural viral clearance. Our study not only addresses these key questions but also contributes to the broader understanding of the evolving SARS-CoV-2 landscape. In an era marked by the coexistence of various viral strains, the need to optimize therapeutic strategies remains paramount. The answers to these questions hold the potential to inform clinical decisions, refine treatment protocols, and ultimately enhance our ability to combat COVID-19, safeguarding public health worldwide. Through analysis and comprehensive data collection, we endeavored to provide critical insights that will shape the future of monoclonal antibody-based interventions in the battle against SARS-CoV-2 and other viral infections.

## SUPPLEMENTARY DATA

Supplementary materials are available at the *Pathogens and Immunity* website. Supplementary data may be provided by the authors to benefit the reader. Supplementary data are not copyedited and are the sole responsibility of the authors. Questions or comments related to supplementary materials should be addressed to the corresponding author.
