# Supplementary material for: Impact of SARS-CoV-2 Resistance to Antiviral Monoclonal Antibody Therapy on Neutralizing Antibody Response
Source: Pathog Immun. 2024 Aug 21;9(2):79–93. doi: 10.20411/pai.v9i2.718 (PMC11378757; doi:10.20411/pai.v9i2.718)
Supplement: Supplementary Figures [file pai-9-079-s01.pdf]

PATHOGENS AND IMMUNITY
IN ASSOCIATION WITH CASE WESTERN RESERVE UNIVERSITY AND THE RICHARD J. FASENMYER FOUNDATION

Supplemental Figure 1

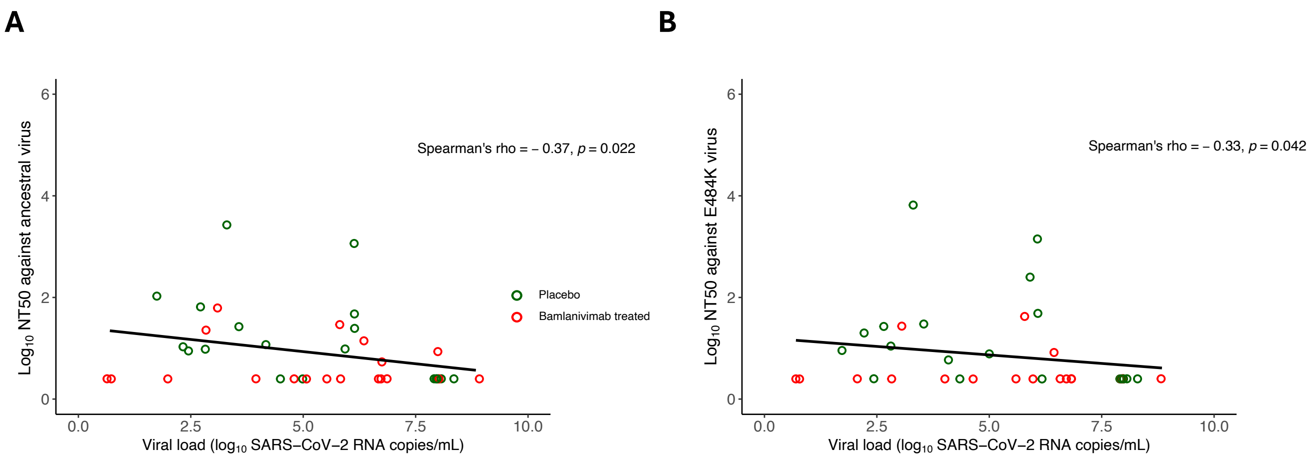

Supplemental Figure 1. Correlation plot of SARS-CoV-2 RNA and NT50 values. (A) against ancestral virus, (B) against E484K virus. Y-axis denotes log_{10} NT50 either against ancestral virus or E484K virus, while x-axis denotes NP SARS-CoV-2 RNA in copies/ml at study entry (day 0). Green and red open circles indicate participants in the placebo and bamlanivimab treatment groups. *P*-values <0.05 are only considered significant and is denoted by *P*, while Spearman's rho is indicated by r.

Paul MK, Choudhary MC, Heaps AL, Deo R, Moisi D, Gordon KC, Mellors JW, Moser C, Klekotka P, Landay A, Currier JS, Eron JJ, Chew KW, Smith DM, Sieg SF, Parikh UM, Li JZ. Impact of SARS-CoV-2 Resistance to Antiviral Monoclonal Antibody Therapy on Neutralizing Antibody Response. *Pathogens and Immunity*. 2024;9(2):79–93. doi: 10.20411/pai.v9i2.718

PATHOGENS AND IMMUNITY
In Association with Case Western Reserve University and The Richard J. Fasenmyer Foundation

Supplemental Figure 2

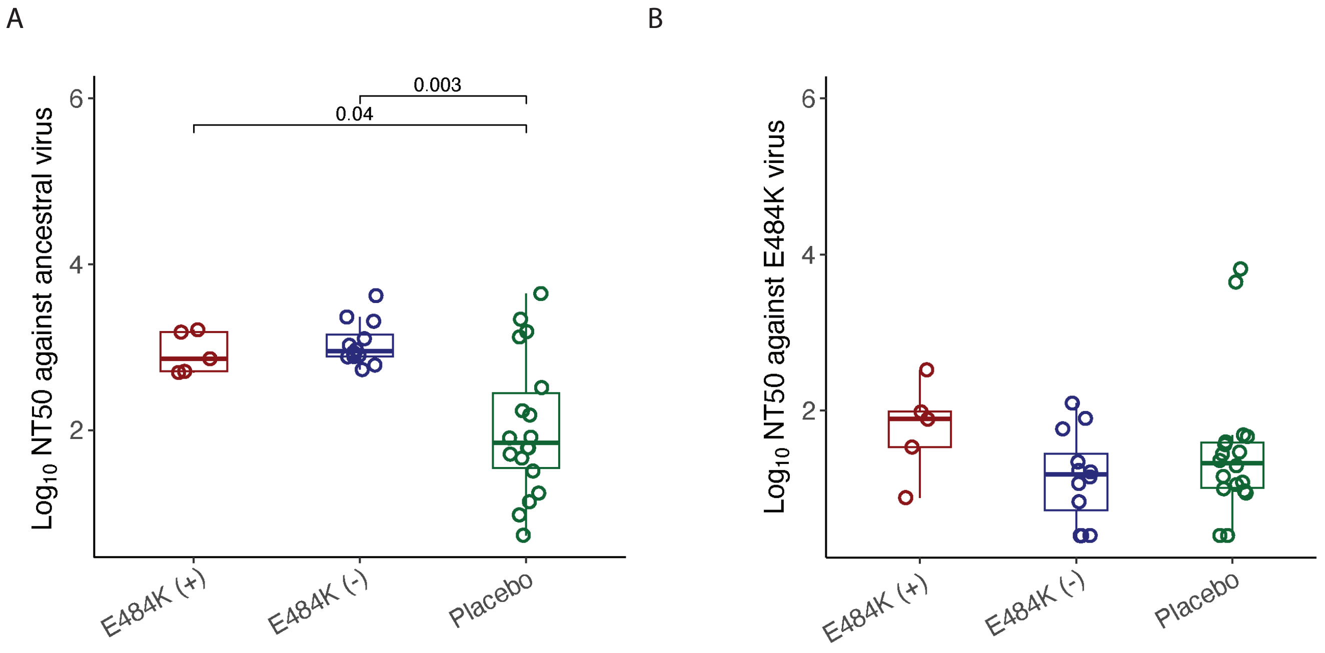

Supplemental Figure 2. Comparison of $\log_{10}$ NT50 values in different study groups at study day 28. (A) against ancestral virus, (B) against E484K virus. The boxplots represent the 25th and 75th percentiles (bottom and top edge of the box), while the open circles represent individual data point. Median values are represented by horizontal line within the boxplot. Comparison of NT50 values between 3 groups were compared using Kruskal-Wallis test. $P<0.05$ were considered statistically significant.

Paul MK, Choudhary MC, Heaps AL, Deo R, Moisi D, Gordon KC, Mellors JW, Moser C, Klekotka P, Landay A, Currier JS, Eron JJ, Chew KW, Smith DM, Sieg SF, Parikh UM, Li JZ. Impact of SARS-CoV-2 Resistance to Antiviral Monoclonal Antibody Therapy on Neutralizing Antibody Response. *Pathogens and Immunity*. 2024;9(2):79–93. doi: 10.20411/pai.v9i2.718
